# New roughage source of *Pennisetum purpureum* cv. Mahasarakham utilization for ruminants feeding under global climate change

**DOI:** 10.5713/ajas.18.0210

**Published:** 2018-05-31

**Authors:** Chaowarit Mapato, Metha Wanapat

**Affiliations:** 1 Tropical Feed Resources Research and Development Center (TROFREC), Department of Animal Science, Faculty of Agriculture, Khon Kaen University, Khon Kaen 40002, Thailand

**Keywords:** Forage Utilization, Sweet Grass, Feed Quality, Global Climate Change, Ruminants

## Abstract

**Objective:**

As the climate changes, it influences ruminant’s feed intake, nutrient digestibility, rumen methane production and emission. This experiment aimed to evaluate the effect of feeding Sweet grass (*Pennisetum purpureum* cv. Mahasarakham; SG) as a new source of good quality forage to improve feed utilization efficiency and to mitigate rumen methane production and emission.

**Methods:**

Four, growing crossbred of Holstein Friesian heifers, 14 months old, were arranged in a 4×4 Latin square design to receive four dietary treatments. Treatment 1 (T1) was rice straw (RS) fed on *ad libitum* with 1.0% body weight (BW) of concentrate (C) supplementation (RS/1.0C). Treatment 2 (T2) and treatment 3 (T3) were SG, fed on *ad libitum* with 1.0% and 0.5% BW of concentrate supplementation, respectively (SG/1.0C and SG/0.5C, respectively). Treatment 4 (T4) was total Sweet grass fed on *ad libitum* basis with non-concentrate supplementation (TSG).

**Results:**

The results revealed that roughage and total feed intake were increased with SG when compared to RS (p<0.01) while TSG was like RS/1.0C treatment. Digestibility of nutrients, nutrients intake, total volatile fatty acids (VFAs), rumen microorganisms were the highest and CH4 was the lowest in the heifers that received SG/1.0C (p<0.01). Total dry matter (DM) feed intake, digestibility and intake of nutrients, total VFAs, NH_3_-N, bacterial and fungal population of animals receiving SG/0.5C were higher than those fed on RS/1.0C. Reducing of concentrate supplementation with SG as a roughage source increased NH_3_-N, acetic acid, and fungal populations, but it decreased propionic acid and protozoal populations (p<0.05). However, ruminal pH and blood urea nitrogen were not affected by the dietary treatments (p>0.05).

**Conclusion:**

As the results, SG could be a good forage to improve rumen fermentation, decrease methane production and reduced the level of concentrate supplementation for growing ruminants in the tropics especially under global climate change.

## INTRODUCTION

As global warming occurs, it has been a topic of concern and significance for all, especially those engaged in ruminant production. Feed resources and feeding manipulation are of paramount importance to improve rumen fermentation efficiency and reduce methane production, which can affect global warming [1,2]. Roughages are a very important feed resource for feed utilization in ruminants. Among numerous sources, rice straw is an abundantly available feed resource in the sub-tropical and tropical area. Its low protein and high cellulose-lignin content can reduce nutrient digestibility, feed intake and production performances. In general, heifers are traditionally fed a high amount of roughage and a moderately low energy diet. However, low quality roughage feeding requires a high level of concentrate supplementation to compensate for the low nutrient intake which would result in decreased feed efficiency of the animal and increased production costs. Roughage has been considered a low cost source of nutrients and grain has been considered expensive, as a result, roughage use often was maximized to reduce feed cost. In addition, the other benefits of roughage included improved ruminal pH, overall rumen health, milk components, and economic return [3–5]. However, the quality of roughage is an important factor affecting rumen fermentation and feed degradation [6]. High quality roughage will allow the rumen microbes to increase the digestion of roughage itself, supplying more nutrients to the animal which could reduce the concentrate supplementation [3]. Furthermore, high quality forage diet feeding can increase the fiber physical effectiveness that could stimulate the cud chewing activity and decrease the risk of ruminal acidosis. Nevertheless, good or high quality roughage is required in order to reduce concentrate supplementation while maintaining the animal performances [4,7].

Previous researchers reported that using rich and locally available feed resources with good nutritive composition, such as cassava hay, corn, forage sorghum and tropical legumes, as the alternative forage could reduce concentrate supplementation with improved feed utilization in ruminants [4,5,8,9]. Currently, Mapato and Wanapat [10] found that Sweet grass (*Pennisetum purpureum* cv. Mahasarakham; SG), a dwarf Napier grass variety, contains a high nutrient composition, especially in crude protein (15.2% dry matter [DM]), non-fiber carbohydrate (NFC, 12.1% DM) and is low in acid detergent fiber (ADF) content (34.9% DM). These characteristics are achieved when the plant is harvested at 42 days of growth when there is a high leaf to stem ratio leading to a high DM and organic matter degradability, and lower *in vitro* gas production when compared with other tropical grasses namely Ruzi, Guinea, and Napier Pakchong1. At a higher ratio of roughage to concentrate (80:20), SG has similar in *in vitro* degradability and gas production to the diet containing Guinea at a 60:40 ratio, in which Guinea grass was the lowest quality while SG was the highest quality forage. It appeared that SG can reduce concentrate supplementation. Nevertheless, more investigation on feed intake, rumen fermentation, and nutrient utilization are still required, especially on feed utilization with reduced concentrate supplementation. Therefore, the aim of this experiment was to determine the use of SG as a good quality forage to reduce the amount of concentrate supplementation in growing crossbred Holstein dairy in hot tropical conditions.

## MATERIALS AND METHODS

### The experimental area

This experiment was performed at the Ruminant Nutrition and Metabolism Research Center, Tropical Feed Resource Research and Development Center (TROFREC), Department of Animal Science, Faculty of Agriculture, Khon Kaen University, Khon Kaen, Thailand. The experimental methods and all animals were allowed by the Khon Kaen University Animal Ethics Committee, ancillary to the Ethic of Animal Experimentation of National Research Council of Thailand.

### Animals, diets and experimental design

Four crossbred Holstein-Frisian heifers (mean±standard deviation) of body weight [BW]; 203±3.2 kg as initial weight); 14 months old were randomly assigned in a 4×4 Latin square design. Heifers received four dietary treatments with different roughage sources and concentrate supplementation levels. Rice straw (RS) and fresh SG were the roughage source and were fed on *ad libitum* basis. Levels of concentrate (C) supplementation were 1.0%, 0.5%, and 0% of BW. Four treatments are as follows: T1 was RS with 1.0% BW of C (RS/1.0C); T2 and T3 were SG with 1.0% and 0.5% BW of C, respectively (SG/1.0C and SG/0.5C, respectively). T4 was total SG fed on *ad libitum* (TSG). Experimental feed ingredients of the concentrate, nutrient composition of all experimental feeds are presented in Table 1. All experimental animals were treated with vitamin A, D_3_, E injection and deworming before imposing the respective treatments.

The experiment was carried out for four periods with each period consisting of 21 days. The first 14 days were for animal adaptation, while the last 7 days for sample collection. Animals were raised in individual pen and received the experimental diets twice a day at 7.00 am and 3.00 pm with free water and mineral lick blocks. Fresh SG was cut around 45 days of growth and chopped daily before feeding. The difference between roughage quantity allocated and refusals were voluntary feed intake of roughages for every day of the trial. Concentrates were fed according to the respective treatments.

### Sampling procedures and experiment analysis methods

All procedures and feed details collection, rumen fluid, blood samples and rumen methane calculation were done according to all procedures reported in Wanapat et al [11]. All samples such as the concentrate, rice straw, SG and fecal samples were taken at every period during the last 7 days. The samples were separated into two parts, the first part was used to analyze for DM. The second part was for crude protein (CP), ether extract (EE) and ash, using the technique of AOAC [12]. Neutral detergent fiber (NDF) and ADF were analyzed by the technique of Van Soest et al [13]. The NFC was calculated according to the equation of NRC [14] which % NFC = 100–(NDF+CP+ EE+Ash).

Rumen fluid was taken at 0 and 4 hours after feeding on the last day of each period. Approximately, 200 mL of rumen fluid from each animal was collected by stomach tube connected to a vacuum pump. Rumen pH was measured immediately by a portable pH meter (HANNA instrument HI 8424 microcomputer, Singapore, Singapore) and then filtered through 4 layers of cheesecloth. Forty-five mL of rumen fluid was mixed with 5 mL of 1 M H_2_SO_4_ and used for concentration of ammonia nitrogen (NH_3_-N) and volatile fatty acids (VFAs) analysis using the Kjeltech Auto 1030 Analyzer [12], and high pressure liquid chromatography instruments by water and Novapak model 600E; water mode 1484UV detector; column Novapak C18; column size 3.9×300 mm; mobile phase 10 mM H_2_PO_4_ (pH 2.5) according to Samuel et al [15], respectively. Prediction of ruminal methane (CH_4_) production using VFAs proportions was made using the equation of Moss et al [16] as follows:

$${\text{CH}}_{4}\;\text{production}=0.45\;(\text{acetate})-0.275\;(\text{propionate})+0.4\;(\text{butyrate})\;\text{unit}$$

Another 1 mL portion was taken and collected in a plastic bottle to which 9 mL of 10% formalin solution was added, it was then kept at 4°C for counting bacteria, fungi and protozoa population using the total direct count technique according to the method of Galyen [17]. Ten mL of Blood were taken from the jugular vein of each animal and immediately kept on ice and transported to the laboratory for analyzing blood urea nitrogen (BUN) using the technique of Crocker [18].

### Statistical analysis

All data were analyzed with analysis of variance according to a 4×4 Latin square design using the general linear models procedures [19]. Data was analyzed using the model Y_ijk_ = μ+M_i_+A_j_+P_k_+ɛ_ijk_, where Y_ijk_ = observation from animal j, receiving diet i, in period k; μ = the overall of the mean; M_i_ = the mean effect of dietary treatments (i = 1, 2, 3, 4); A_j_ = the effect of animal (j = 1, 2, 3, 4); P_k_ = the effect of period (k = 1, 2, 3, 4); and ɛ_ijk_ = the residual error. The results were presented as mean values with the standard error of the means. Differences between means with p<0.05 was accepted as statistical differences which treatment means were determined by using the Duncan’s new multiple range test [20].

## RESULTS

### Chemical composition of the experimental diets

Feed ingredients, nutrient composition of concentrate and roughages are shown in Table 1. Concentrates were formulated and mixed using local feed ingredients to contain 14.1% CP. Rice straw has a lower CP (2.8%) and higher NDF and ADF content (75.8% and 46.8%, respectively). The SG has a greater content of CP and NFC contents (14.7% and 15.2%, respectively) while it has a lower content of NDF and ADF concentration (55.7% and 33.1%, respectively). In addition, SG contains high level of NFC (15.2%).

### Voluntary feed intake, digestibility and intake of nutrients

Voluntary feed intake of roughages was different among dietary treatments (p<0.01) while ratios of roughage intake were increased (55.8%, 64.1%, 81.0%, and 100.0% of DM, respectively) when reducing concentrate supplementation levels (Table 2). Concentrate supplementation at 0.5% BW with SG showed a greater feed intake, nutrient digestibility and nutrients intake compared to 1.0% BW concentrate fed with rice straw as the control group. Nutrients digestibility and intake of nutrients were higher with SG feeding when compared to rice straw feeding at the same concentrate supplementation at 1.0% of BW (p<0.01). However, there were no significant differences among treatments in the ADF digestibility and ADF intake (p>0.05). SG improved the nutrients intake and digestibility when compared to RS. Heifers that received the SG/1.0C diet had a greater total feed intake, digestibility, and nutrients intake while SG/0.5C and TSG were better but similar to RS/1.0C, respectively.

### Rumen fermentation characteristics and blood metabolites

Table 3 shows that acetic acid, NH_3_-N concentration and fungi population were increased with decreasing concentrate supplementation (p<0.05) while it decreased propionic acid and protozoal numbers (p<0.05). Propionic acid, total VFAs, and bacterial population were the highest values with heifer receiving SG/1.0C diet (p<0.05). The CH4 production was decreased with SG/1.0C as compared to RS/1.0C diet (p<0.05). The TSG group has the highest value of acetic acid, NH_3_-N and fungal population while they have the lowest protozoal population (p<0.05). Ruminal pH, butyric acid concentration and BUN levels were not affected by the experimental diets (p>0.05).

## DISCUSSION

### Chemical composition of the experimental diets

RS was lower in CP and higher in fibrous contents compared to SG, especially the CP and carbohydrate contents (higher in NFC and lower NDF, ADF contents). This is due to it being a leafier grass and having a higher leaf to stem ratio that reflected on higher nutritive values. Other using dwarfs Napier grass with leafier grass, also showed high nutritive values [10,21–24].

### Voluntary feed intake, digestibility and intake of nutrients

As the result of feed intake, concentrate could be fed at 0.5% BW with SG-based when compared to 1.0% BW with RS-based in heifer feeding. It appears that SG could reduce the use of concentrate supplementation without negative effect on the amount of feed uptake, nutrient digestibility and intake of nutrients. These results are in accordance with previous experiments which showed that SG was a high quality roughage, especially as it has the lowest content of NDF and ADF when compared to Ruzi, Guinea and Napier Pakchong1 grass at a similar age of regrowth. This resulted in having the highest degradability and accumulated gas production, using *in vitro* gas production technique. Moreover, it was found that higher quality roughage could decrease levels of concentrate supplementation [10]. The results of this experiment confirmed that voluntary feed intake of roughage, nutrient digestibility and nutrients intake were significantly related to the quality of roughage as NRC, [14] presented that NDF and ADF content were highly related to DM intake and digestibility of roughage. The current results were according to those of previous studies which showed feeding higher quality roughage could increase feed efficiency in ruminants [5,24–26]. Corea et al [26] also found that using Cow-pea as a high quality roughage could reduce the CP content in concentrate from 17.0% to 15.5% without altering the feed digestibility, milk production, and milk components of lactating dairy cows. It also agreed with the findings of Wanapat et al [5] that higher quality roughage achieved using the tropical legume (*Phaseolus calcaratus*) mixed with Ruzi grass could improve DM intake, nutrient digestibility and milk production. Moreover, it could be used to reduce the amount of concentrate supplementation without adversely effect on rumen fermentation characteristics and milk production in tropical dairy cows. Therefore, quality of roughage is very important to the improvement of feed intake and feed utilization efficiency in ruminant feeding to ensure efficient rumen fermentation and production.

### Rumen fermentation characteristics and blood metabolites

Rumen fermentation parameters such as pH, NH_3_-N, VFAs, CH_4_, rumen microbe population and BUN were measured to determine the relationship between the dietary treatments and rumen ecology. As the results, rumen pH and NH_3_-N were within normal range of 6.3 to 7.2 and 15 to 30 mg %, respectively [27–29]. However, NH_3_-N tended to increase with the increasing of SG intake. It was found that the utilization of high protein forage needs carbohydrate (starch and sugar) to be synchronized for efficient microbial protein production with a decrease in NH_3_-N concentration [30]. In this experiment, it was found that reducing carbohydrate supply from concentrates resulted in increased NH_3_-N. The feeding of SG/0.5C showed similar response in these parameters with RS/1.0C. Using higher quality roughage provided more nutrients to rumen microbes for growth and resulted in increased microbial population and rumen fermentation end-products expressed in total VFAs and propionic acid concentration. Total VFA, propionic acid, bacterial and protozoal populations were highest in the heifers which received SG/1.0C as related with feed intake and digestibility as compared with RS/1.0C. Moreover, it could decrease CH4 emission that might be due to the nutrient composition of roughage influencing on methane yield, particularly hexose fermented and VFAs during the fermentation process in the rumen. Increasing of propionate with decreasing of acetate and butyrate by increasing of NFC in the diet resulted in decreased methane production. Forages that have a high content of NFC, generally support fermentation that increase production of propionate, which competes for hydrogen with methane production. Moreover, forages that have less NDF content could increase digestibility resulting in producing less methane [1,31,32].

Nevertheless, it was found that decreasing concentrate intake would decrease the protozoal population. The number of protozoa was related to level of concentrate feeding with reduction of concentrate leading to decreased protozoal population. Jouany [32] reported that dietary factors, such as roughage to concentrate ratio, forage quality and level of feeding influenced protozoal populations. Another experiment using cassava hay as high quality roughage to reduce concentrate supplementation resulted in improved rumen ecology, particularly increased rumen bacterial and decreased protozoal population, which was associated with enhanced feeds digestibility, milk yield and milk components in dairy cows [8]. This agrees with Martinez et al [33] who found that in sheep a high ratio of concentrate feeding (30:70, R:C ratio) increased total number of protozoa as compared to a low ratio of concentrate (70:30, R:C ratio). In this case, reducing concentrate supplementation and therefore reducing starch and sugar that are the main feed resources for protozoa resulted in decreased protozoal growth. In addition, the populations of fungi were increased by reducing of concentrate supplementation due to increasing the roughage intake improved growing conditions of fungi. Kamra et al [34] reported that the fungal growth was stimulated by the high fiber diets of buffalo when compared to high concentrate diets which rich in non-structural carbohydrates. These numbers of fungi propose to get adhered to the feed particle and related to the digestion efficiency in ruminants [35]. Therefore, it could explain why the highest numbers of fungi were associated with the total SG feeding, with better NDF digestibility than an RS/1.0C. The current study revealed that rumen microorganism populations were highly related to nutrient digestibility, which has a significant role in feed degradation in the rumen, particularly bacterial and fungal populations [36,37]. Moreover, this study suggests that feeding of concentrate supplementation at 0.5% BW with good quality roughage would support efficient rumen fermentation as resulted in the fermentation end-products.

## CONCLUSION

In conclusion, 1.0% BW of concentrate supplementation with SG improves feed utilization and rumen fermentation. Higher quality roughage could reduce concentrate supplementation when compared to lower quality roughage. High level of roughage feeding, in which 0.5% BW of concentrate supplementation with SG is recommended. Nevertheless, future studies should be conducted provide more data on its feeding value to produce milk and meat.

## Figures and Tables

**Table 1:** Feed ingredients of the concentrate and chemical composition of the feeds

Items	Concentrate	Sweet grass	Rice straw
Ingredients (%, fresh basis)			
Cassava chip	60.0	-	-
Rice bran	10.0	-	-
Coconut meal	15.0	-	-
Palm meal	10.0	-	-
Urea	1.5	-	-
Molasses	2.0	-	-
Sulfur	0.5	-	-
Mineral mixture	0.5	-	-
Salt	0.5	-	-
Total	100.0	-	-
Chemical composition			
Dry matter (%)	91.1	13.2	92.7
	--------------------% of dry matter-----------------		
Crude protein	14.1	14.7	2.8
Ether extract	2.8	2.3	1.4
Acid detergent fiber	12.9	33.1	46.8
Neutral detergent fiber	18.7	55.7	75.8
Ash	7.5	9.8	16.3
Non fiber carbohydrate	52.9	15.2	3.7

**Table 2:** Effect of dietary treatments on feed intake, nutrient digestibility and nutrient intake in dairy heifers

Items	Treatments1)				SEM

	RS/1.0C	SG/1.0C	SG/0.5C	TSG	
Roughage intake					
kg of DM/d	2.9^d^	4.1^c^	4.7^b^	5.2^a^	0.05
% of BW	1.4^d^	1.9^c^	2.1^b^	2.4^a^	0.02
Concentrate intake					
kg of DM/d	2.2	2.2	1.1	-	-
% of BW	1.0	1.0	0.5	-	-
Total feed intake					
kg of DM/d	5.2^c^	6.4^a^	5.8^b^	5.2^c^	0.05
% of BW	2.4^c^	2.9^a^	2.6^b^	2.4^c^	0.02
Nutrient digestibility (%)					
Dry matter	64.2^c^	70.2^a^	66.5^b^	62.5^d^	0.21
Organic matter	64.0^c^	70.8^a^	67.6^b^	63.6^c^	0.12
Crude protein	66.4^b^	79.7^a^	78.3^a^	77.1^a^	0.40
Neutral detergent fiber	57.5^d^	65.3^a^	63.0^b^	61.3^c^	0.11
Acid detergent fiber	51.8^b^	56.1^a^	55.0^a^	52.7^b^	0.17
Nutrient intake (kg/d)					
Dry matter	3.3^c^	4.5^a^	3.9^b^	3.3^c^	0.04
Organic matter	2.9^c^	4.1^a^	3.6^b^	3.0^c^	0.04
Crude protein	0.3^b^	0.7^a^	0.7^a^	0.6^a^	0.01
Neutral detergent fiber	1.5^b^	1.8^a^	1.8^a^	1.8^a^	0.01
Acid detergent fiber	0.9	0.9	0.9	0.9	0.002

RS, rice straw; SG, sweet grass; TSG, total sweet grass; SEM, standard error of the means; DM, dry matter; BW, body weight.

1) RS/1.0C, RS fed on *ad libitum*+1.0% BW of concentrate; SG/1.0C, SG fed on *ad libitum*+1.0% BW of concentrate; SG/0.5C, SG fed on *ad libitum*+0.5% BW of concentrate; TSG, total SG fed on *ad libitum*.

abcd different among treatment means (p<0.05).

**Table 3:** Effect of dietary treatments on rumen fermentation and blood urea nitrogen concentration in dairy heifers

Items	Treatments1)				SEM

	RS/1.0C	SG/1.0C	SG/0.5C	TSG	
Ruminal pH	6.3	6.5	6.5	6.6	0.14
Total VFAs (mg/dL)	97.0^b^	100.0^a^	99.5^a^	91.6^c^	0.14
Molar of VFAs (%)					
Acetic acid	66.5^b^	63.3^c^	66.9^b^	68.6^a^	0.26
Propionic acid	24.1^b^	27.0^a^	23.7^b^	21.9^c^	0.22
Butyric acid	9.4	9.7	9.4	9.5	0.04
CH_4_ (mmol/100 mol)	27.1^b^	24.9^a^	27.3^b^	28.6^b^	0.21
Ammonia-nitrogen (mg%)	21.2^c^	20.4^c^	25.1^b^	29.7^a^	0.47
Blood urea nitrogen (mg/dL)	13.9	14.5	14.4	15.5	0.51
Rumen microbe population (cell/mL)					
Bacteria (×10^11^)	30.8^c^	37.5^a^	34.2^b^	30.3^c^	0.76
Fungi (×10^6^)	2.5^c^	5.0^b^	6.5^ab^	8.0^a^	0.35
Protozoa (×10^5^)	10.8^ab^	11.3^a^	8.3^b^	6.0^c^	0.69

RS, rice straw; SG, sweet grass; TSG, total sweet grass; SEM, standard error of the means; VFAs, volatile fatty acids; BW, body weight.

1) RS/1.0C, RS fed *ad libitum*+1.0% BW of concentrate; SG/1.0C, SG fed *ad libitum*+1.0% BW of concentrate; SG/0.5C, SG fed *ad libitum*+0.5% BW of concentrate; TSG, total SG fed *ad libitum*.

abc Different among treatment means (p<0.05).
